# Supplementary material for: Systematic identification of smORFs in domestic silkworm (Bombyx mori)
Source: PeerJ. 2023 Jan 13;11:e14682. doi: 10.7717/peerj.14682 (PMC9841908; doi:10.7717/peerj.14682)
Supplement: Supplemental Information 1 [file peerj-11-14682-s001.doc]

Table S1. Collection of silkworm transcriptome sequencing datasets.

| Abbreviations | Tissues | SAR Accessions | References |
| --- | --- | --- | --- |
| ASG | Anterior silk gland | DRR186474,DRR186475,DRR186476 | [1] |
| MSG_A | Anterior part of the middle silk gland | DRR186477,DRR186478,DRR186479 | [1] |
| MSG_M | Middle part of the middle silk gland | DRR186480,DRR186481,DRR186482 | [1] |
| MSG_P | Posterior part of the middle silk gland | DRR186483,DRR186484,DRR186485 | [1] |
| PSG | Posterior silk gland | DRR186486,DRR186487,DRR186488 | [1] |
| FB | Fat body | DRR186489,DRR186490,DRR186491 | [1] |
| MG | Midgut | DRR186492,DRR186493,DRR186494 | [1] |
| MT | Malpighian tuble | DRR186495,DRR186496,DRR186497 | [1] |
| TT | Testis | DRR186498,DRR186499,DRR186500 | [1] |
| OV | Ovary | DRR186501,DRR186502,DRR186503 | [1] |
| BN_TT | Testis | DRR068893,DRR068894,DRR068895 | [2,3] |
| BN_FB | Fat body | DRR095105,DRR095106,DRR095107 | [2] |
| BN_MG | Midgut | DRR095108,DRR095109,DRR095110 | [2,4] |
| BN_MT | Malpighian tuble | DRR095111,DRR095112,DRR095113 | [2] |
| BN_SG | Silk gland | DRR095114,DRR095115,DRR095116 | [2] |

**References**

1. Yokoi, K.; Tsubota, T.; Jouraku, A.; Sezutsu, H.; Bono, H. Reference Transcriptome Data in Silkworm Bombyx mori. *Insects* **2021**, *12*, 519.

2. Kobayashi, Y.; Nojima, Y.; Sakamoto, T.; Iwabuchi, K.; Nakazato, T.; Bono, H.; Toyoda, A.; Fujiyama, A.; Kanost, M.R.; Tabunoki, H. Comparative analysis of seven types of superoxide dismutases for their ability to respond to oxidative stress in Bombyx mori. *Sci. Rep.-Uk* **2019**, *9*, 2170, doi: 10.1038/s41598-018-38384-8.

3. Kikuchi, A.; Nakazato, T.; Ito, K.; Nojima, Y.; Yokoyama, T.; Iwabuchi, K.; Bono, H.; Toyoda, A.; Fujiyama, A.; Sato, R.; et al. Identification of functional enolase genes of the silkworm Bombyx mori from public databases with a combination of dry and wet bench processes. *Bmc Genomics* **2017**, *18*, 83.

4. Ichino, F.; Bono, H.; Nakazato, T.; Toyoda, A.; Fujiyama, A.; Iwabuchi, K.; Sato, R.; Tabunoki, H. Construction of a simple evaluation system for the intestinal absorption of an orally administered medicine using *Bombyx mori* larvae. *Drug Discoveries & Therapeutics* **2018**, *12*, 7-15, doi: 10.5582/ddt.2018.01004.

Table S2. Classification criteria for each confidence level.

| Classification levels | Sources | | | |
| --- | --- | --- | --- | --- |
|  | with homologous in the database | CPPred-sORF | With de novo transcript evidence | With de novo evolutionary evidence |
| HC (High Confidence) | | | | |
| HC1 | **+** | **+** | **+** | **+** |
| HC2 | **+** | **-** | **+** | **+** |
| HC3 | **-** | **+** | **+** | **+** |
| HC4 | **-** | **-** | **+** | **+** |
| LC (How Confidence) | | | | |
| LC1 | **-** | **-** | **+** | **-** |
| LC2 | **-** | **-** | **-** | **+** |

Table S3. Definitions of each smORF type.

| Types | Definitions |
| --- | --- |
| 1. inFrameORF | located in protein-coding genes, with same frame |
| 1. outFrameORF | located in protein-coding genes, with different frame |
| 2.1 uORF | located in upstream of genes |
| 2.2 dORF | located in the downstream of genes |
| 2.3 ncORF | Located in ncRNA genes |
| 2.3.1 lncORF | Located in lncRNA genes |
| 2.3.2 miORF | Located in miRNA genes |
| 2.3.3 circORF | Located in circRNA genes |
| 1. intronORF | located in introns |
| 1. intergenicORF | located in the intergenic regions |

Table S4. Statistics of sequencing reads datasets before and after filtering.

| Sample | Raw reads | Raw bases | Clean reads | Clean bases | Reads retention ratio (%) |
| --- | --- | --- | --- | --- | --- |
| BN_TT-1 | 33,459,860 | 5,018,979,000 | 21,377,656 | 2,950,653,449 | 63.89 |
| BN_TT-2 | 33,766,152 | 5,064,922,800 | 21,557,396 | 2,974,613,652 | 63.84 |
| BN_TT-3 | 21,697,584 | 3,254,637,600 | 14,451,914 | 2,009,067,607 | 66.61 |
| BN_FB-1 | 20,526,444 | 3,078,966,600 | 12,952,478 | 1,801,721,104 | 63.1 |
| BN_FB-2 | 31,092,124 | 4,663,818,600 | 18,797,716 | 2,595,353,386 | 60.46 |
| BN_FB-3 | 31,322,066 | 4,698,309,900 | 18,926,584 | 2,612,421,669 | 60.43 |
| BN_MG-1 | 21,225,604 | 3,183,840,600 | 12,983,302 | 1,798,758,848 | 61.17 |
| BN_MG-2 | 32,241,798 | 4,836,269,700 | 18,777,972 | 2,580,403,307 | 58.24 |
| BN_MG-3 | 32,457,226 | 4,868,583,900 | 18,883,572 | 2,594,005,816 | 58.18 |
| BN_MT-1 | 21,771,964 | 3,265,794,600 | 14,440,160 | 2,006,422,065 | 66.32 |
| BN_MT-2 | 33,029,924 | 4,954,488,600 | 21,027,440 | 2,901,673,638 | 63.66 |
| BN_MT-3 | 33,296,022 | 4,994,403,300 | 21,188,230 | 2,923,269,123 | 63.64 |
| BN_SG-1 | 21,169,266 | 3,175,389,900 | 12,454,914 | 1,730,090,656 | 58.83 |
| BN_SG-2 | 32,830,332 | 4,924,549,800 | 18,636,652 | 2,571,842,276 | 56.77 |
| BN_SG-3 | 33,152,906 | 4,972,935,900 | 18,813,148 | 2,595,528,483 | 56.75 |
| ASG-1 | 30,487,082 | 3,079,195,282 | 29,615,348 | 2,681,322,729 | 97.14 |
| ASG-2 | 41,678,278 | 4,209,506,078 | 40,569,074 | 3,674,694,546 | 97.34 |
| ASG-3 | 40,987,262 | 4,139,713,462 | 39,967,412 | 3,620,896,374 | 97.51 |
| MSG_A-1 | 43,337,486 | 4,377,086,086 | 42,331,822 | 3,833,054,562 | 97.68 |
| MSG_A-2 | 32,080,426 | 3,240,123,026 | 31,045,744 | 2,807,809,065 | 96.77 |
| MSG_A-3 | 42,298,382 | 4,272,136,582 | 41,204,618 | 3,730,225,789 | 97.41 |
| MSG_M-1 | 35,459,624 | 3,581,422,024 | 34,366,660 | 3,110,485,399 | 96.92 |
| MSG_M-2 | 42,473,172 | 4,289,790,372 | 41,218,904 | 3,730,205,210 | 97.05 |
| MSG_M-3 | 34,033,404 | 3,437,373,804 | 33,002,980 | 2,986,911,772 | 96.97 |
| MSG_P-1 | 37,724,178 | 3,810,141,978 | 36,666,072 | 3,319,499,151 | 97.2 |
| MSG_P-2 | 43,993,028 | 4,443,295,828 | 42,676,594 | 3,863,686,812 | 97.01 |
| MSG_P-3 | 33,293,664 | 3,362,660,064 | 32,442,208 | 2,937,201,182 | 97.44 |
| PSG-1 | 41,092,186 | 4,150,310,786 | 39,986,768 | 3,621,406,804 | 97.31 |
| PSG-2 | 43,945,372 | 4,438,482,572 | 42,713,302 | 3,867,756,360 | 97.2 |
| PSG-3 | 32,952,544 | 3,328,206,944 | 31,978,094 | 2,894,990,190 | 97.04 |
| FB-1 | 42,980,928 | 4,341,073,728 | 41,900,576 | 3,796,244,092 | 97.49 |
| FB-2 | 33,091,308 | 3,342,222,108 | 32,127,150 | 2,909,616,765 | 97.09 |
| FB-3 | 50,554,656 | 5,106,020,256 | 49,490,304 | 4,487,285,256 | 97.89 |
| MG-1 | 34,737,884 | 3,508,526,284 | 33,703,250 | 3,051,821,105 | 97.02 |
| MG-2 | 38,501,946 | 3,888,696,546 | 37,495,480 | 3,395,971,543 | 97.39 |
| MG-3 | 40,627,880 | 4,103,415,880 | 39,317,288 | 3,559,222,779 | 96.77 |
| MT-1 | 43,767,422 | 4,420,509,622 | 42,690,618 | 3,870,656,225 | 97.54 |
| MT-2 | 33,500,080 | 3,383,508,080 | 32,114,498 | 2,911,943,734 | 95.86 |
| MT-3 | 40,006,180 | 4,040,624,180 | 37,898,364 | 3,436,083,534 | 94.73 |
| TT-1 | 37,206,746 | 3,757,881,346 | 35,148,070 | 3,186,833,510 | 94.47 |
| TT-2 | 42,344,850 | 4,276,829,850 | 39,945,082 | 3,622,401,011 | 94.33 |
| TT-3 | 43,515,646 | 4,395,080,246 | 41,314,354 | 3,745,789,380 | 94.94 |
| OV-1 | 43,773,604 | 4,421,134,004 | 41,638,262 | 3,775,226,663 | 95.12 |
| OV-2 | 51,966,226 | 5,248,588,826 | 49,656,404 | 4,502,996,478 | 95.56 |
| OV-3 | 34,909,946 | 3,525,904,546 | 33,174,172 | 3,006,984,420 | 95.03 |

Table S5. Alignment rates for each sample.

| Sample Name | total_reads | overall_align_rate | aligned_1_time |
| --- | --- | --- | --- |
| ASG-1 | 14,807,674 | 98.44% | 87.61% |
| ASG-2 | 20,284,537 | 98.68% | 87.90% |
| ASG-3 | 19,983,706 | 98.53% | 87.86% |
| BN_FB-1 | 6,476,239 | 95.21% | 64.18% |
| BN_FB-2 | 9,398,858 | 95.41% | 64.32% |
| BN_FB-3 | 9,463,292 | 95.40% | 64.15% |
| BN_MG-1 | 6,491,651 | 94.23% | 49.49% |
| BN_MG-2 | 9,388,986 | 94.39% | 49.10% |
| BN_MG-3 | 9,441,786 | 94.37% | 49.59% |
| BN_MT-1 | 7,220,080 | 90.60% | 52.25% |
| BN_MT-2 | 10,513,720 | 90.95% | 52.02% |
| BN_MT-3 | 10,594,115 | 90.99% | 51.65% |
| BN_SG-1 | 6,227,457 | 85.70% | 43.37% |
| BN_SG-2 | 9,318,326 | 85.69% | 43.58% |
| BN_SG-3 | 9,406,574 | 85.65% | 43.27% |
| BN_TT-1 | 10,688,828 | 90.29% | 57.89% |
| BN_TT-2 | 10,778,698 | 90.24% | 57.46% |
| BN_TT-3 | 7,225,957 | 90.11% | 57.99% |
| FB-1 | 20,950,288 | 97.81% | 86.66% |
| FB-2 | 16,063,575 | 99.27% | 87.59% |
| FB-3 | 24,745,152 | 98.95% | 88.45% |
| MG-1 | 16,851,625 | 99.15% | 77.78% |
| MG-2 | 18,747,740 | 99.15% | 78.80% |
| MG-3 | 19,658,644 | 99.04% | 78.01% |
| MSG_A-1 | 21,165,911 | 97.94% | 80.66% |
| MSG_A-2 | 15,522,872 | 98.07% | 79.54% |
| MSG_A-3 | 20,602,309 | 94.18% | 77.51% |
| MSG_M-1 | 17,183,330 | 97.24% | 84.32% |
| MSG_M-2 | 20,609,452 | 98.68% | 82.37% |
| MSG_M-3 | 16,501,490 | 98.48% | 84.07% |
| MSG_P-1 | 18,333,036 | 98.85% | 85.26% |
| MSG_P-2 | 21,338,297 | 98.34% | 84.41% |
| MSG_P-3 | 16,221,104 | 98.76% | 84.20% |
| MT-1 | 21,345,309 | 98.98% | 85.42% |
| MT-2 | 16,057,249 | 99.04% | 84.79% |
| MT-3 | 18,949,182 | 98.92% | 84.26% |
| OV-1 | 20,819,131 | 98.29% | 85.14% |
| OV-2 | 24,828,202 | 98.56% | 85.83% |
| OV-3 | 16,587,086 | 98.53% | 85.93% |
| PSG-1 | 19,993,384 | 98.07% | 84.70% |
| PSG-2 | 21,356,651 | 97.93% | 84.61% |
| PSG-3 | 15,989,047 | 98.83% | 85.10% |
| TT-1 | 17,574,035 | 98.77% | 86.57% |
| TT-2 | 19,972,541 | 98.66% | 85.40% |
| TT-3 | 20,657,177 | 98.53% | 86.69% |

Table S6. Statistics of the assembled transcripts in each sample.

| Sample Name | # novel genes | # novel transcripts | # total genes | # total transcripts | Rate of novel genes | Rate of novel trancripts |
| --- | --- | --- | --- | --- | --- | --- |
| ASG-1 | 3,037 | 4,553 | 9,501 | 11,282 | 31.97% | 40.36% |
| ASG-2 | 3,230 | 5,112 | 10,129 | 12,305 | 31.89% | 41.54% |
| ASG-3 | 3,205 | 5,312 | 10,297 | 12,750 | 31.13% | 41.66% |
| BN_FB-1 | 1,821 | 2,225 | 5,518 | 6,004 | 33.00% | 37.06% |
| BN_FB-2 | 2,536 | 3,057 | 6,664 | 7,283 | 38.06% | 41.97% |
| BN_FB-3 | 2,586 | 3,150 | 6,748 | 7,401 | 38.32% | 42.56% |
| BN_MG-1 | 2,400 | 2,809 | 6,223 | 6,706 | 38.57% | 41.89% |
| BN_MG-2 | 3,294 | 3,834 | 7,727 | 8,354 | 42.63% | 45.89% |
| BN_MG-3 | 3,303 | 3,865 | 7,689 | 8,346 | 42.96% | 46.31% |
| BN_MT-1 | 4,514 | 5,530 | 10,003 | 11,182 | 45.13% | 49.45% |
| BN_MT-2 | 5,822 | 7,150 | 11,789 | 13,320 | 49.39% | 53.68% |
| BN_MT-3 | 5,860 | 7,249 | 11,840 | 13,421 | 49.49% | 54.01% |
| BN_SG-1 | 4,318 | 4,892 | 7,603 | 8,220 | 56.79% | 59.51% |
| BN_SG-2 | 5,355 | 6,118 | 9,049 | 9,865 | 59.18% | 62.02% |
| BN_SG-3 | 5,374 | 6,199 | 9,097 | 9,966 | 59.07% | 62.20% |
| BN_TT-1 | 7,496 | 10,431 | 14,305 | 17,596 | 52.40% | 59.28% |
| BN_TT-2 | 7,447 | 10,447 | 14,267 | 17,605 | 52.20% | 59.34% |
| BN_TT-3 | 6,009 | 8,364 | 12,316 | 14,972 | 48.79% | 55.86% |
| FB-1 | 2,301 | 3,607 | 8,608 | 10,166 | 26.73% | 35.48% |
| FB-2 | 1,969 | 2,920 | 8,174 | 9,355 | 24.09% | 31.21% |
| FB-3 | 2,586 | 4,140 | 9,425 | 11,283 | 27.44% | 36.69% |
| MG-1 | 2,151 | 3,444 | 8,460 | 10,008 | 25.43% | 34.41% |
| MG-2 | 2,486 | 3,932 | 8,960 | 10,687 | 27.75% | 36.79% |
| MG-3 | 2,419 | 3,989 | 9,144 | 11,010 | 26.45% | 36.23% |
| MSG_A-1 | 2,764 | 4,225 | 9,009 | 10,724 | 30.68% | 39.40% |
| MSG_A-2 | 2,525 | 3,732 | 8,548 | 9,979 | 29.54% | 37.40% |
| MSG_A-3 | 2,958 | 4,836 | 9,726 | 11,894 | 30.41% | 40.66% |
| MSG_M-1 | 2,917 | 4,690 | 9,147 | 11,192 | 31.89% | 41.90% |
| MSG_M-2 | 2,973 | 4,819 | 9,415 | 11,542 | 31.58% | 41.75% |
| MSG_M-3 | 3,047 | 4,965 | 9,703 | 11,924 | 31.40% | 41.64% |
| MSG_P-1 | 2,941 | 4,826 | 9,297 | 11,457 | 31.63% | 42.12% |
| MSG_P-2 | 3,103 | 4,909 | 9,468 | 11,546 | 32.77% | 42.52% |
| MSG_P-3 | 2,846 | 4,711 | 9,521 | 11,679 | 29.89% | 40.34% |
| MT-1 | 3,913 | 5,945 | 10,736 | 13,082 | 36.45% | 45.44% |
| MT-2 | 3,677 | 5,562 | 10,558 | 12,764 | 34.83% | 43.58% |
| MT-3 | 4,534 | 6,887 | 11,741 | 14,443 | 38.62% | 47.68% |
| OV-1 | 6,814 | 10,730 | 15,469 | 19,892 | 44.05% | 53.94% |
| OV-2 | 6,025 | 10,038 | 14,799 | 19,335 | 40.71% | 51.92% |
| OV-3 | 6,069 | 9,285 | 14,467 | 18,137 | 41.95% | 51.19% |
| PSG-1 | 2,589 | 4,204 | 8,721 | 10,584 | 29.69% | 39.72% |
| PSG-2 | 2,566 | 4,085 | 8,548 | 10,304 | 30.02% | 39.64% |
| PSG-3 | 2,623 | 4,126 | 8,895 | 10,670 | 29.49% | 38.67% |
| TT-1 | 6,357 | 10,367 | 14,594 | 19,178 | 43.56% | 54.06% |
| TT-2 | 7,054 | 11,517 | 15,547 | 20,615 | 45.37% | 55.87% |
| TT-3 | 6,347 | 10,995 | 14,977 | 20,248 | 42.38% | 54.30% |

Table S7. Statistics of number of alternative splicing isofroms in each sample.

| #Isoform | 1 | 2 | 3 | 4 | 5 | 6 | 7 | 8 | 9 | >=10 |
| --- | --- | --- | --- | --- | --- | --- | --- | --- | --- | --- |
| combined | 17,659 | 3,655 | 1,764 | 738 | 323 | 131 | 79 | 43 | 20 | 30 |
| ASG-1 | 8,203 | 969 | 247 | 59 | 10 | 6 | 3 | 2 | - | 2 |
| ASG-2 | 8,592 | 1,125 | 293 | 88 | 16 | 6 | 5 | 1 | - | 3 |
| ASG-3 | 8,584 | 1,225 | 351 | 89 | 24 | 15 | 3 | 3 | 2 | 1 |
| BN_FB-1 | 5,187 | 246 | 55 | 19 | 5 | 1 | 2 | 1 | 1 | 1 |
| BN_FB-2 | 6,226 | 339 | 70 | 17 | 5 | - | 1 | - | 2 | 4 |
| BN_FB-3 | 6,304 | 331 | 81 | 15 | 6 | 2 | 1 | 2 | 3 | 3 |
| BN_MG-1 | 5,864 | 287 | 44 | 20 | 4 | - | 1 | 1 | 1 | 1 |
| BN_MG-2 | 7,261 | 363 | 76 | 15 | 6 | 1 | - | 2 | 3 | - |
| BN_MG-3 | 7,199 | 383 | 79 | 13 | 9 | 1 | 1 | 2 | 2 | - |
| BN_MT-1 | 9,147 | 644 | 149 | 44 | 11 | 4 | - | - | 2 | 2 |
| BN_MT-2 | 10,642 | 887 | 196 | 45 | 10 | 2 | 3 | 2 | 1 | 1 |
| BN_MT-3 | 10,685 | 867 | 223 | 36 | 21 | 2 | - | 1 | 3 | 2 |
| BN_SG-1 | 7,143 | 371 | 64 | 10 | 7 | 3 | - | 1 | 3 | 1 |
| BN_SG-2 | 8,433 | 497 | 80 | 25 | 6 | 1 | 3 | 1 | 1 | 2 |
| BN_SG-3 | 8,466 | 495 | 91 | 24 | 10 | 4 | 3 | 1 | - | 3 |
| BN_TT-1 | 12,094 | 1,508 | 483 | 145 | 49 | 16 | 5 | 2 | 1 | 2 |
| BN_TT-2 | 12,012 | 1,549 | 487 | 137 | 54 | 19 | 4 | 2 | 2 | 1 |
| BN_TT-3 | 10,463 | 1,334 | 356 | 107 | 36 | 11 | 4 | 2 | 1 | 2 |
| FB-1 | 7,452 | 873 | 221 | 41 | 11 | 4 | 2 | - | - | 4 |
| FB-2 | 7,283 | 689 | 158 | 29 | 5 | 4 | 4 | - | - | 2 |
| FB-3 | 8,092 | 967 | 288 | 47 | 18 | 6 | 2 | - | 2 | 3 |
| MG-1 | 7,300 | 894 | 196 | 48 | 13 | 3 | 2 | - | 2 | 2 |
| MG-2 | 7,658 | 1,001 | 233 | 46 | 11 | 6 | 1 | 1 | - | 3 |
| MG-3 | 7,800 | 987 | 266 | 61 | 19 | 5 | 1 | 1 | 1 | 3 |
| MSG_A-1 | 7,777 | 914 | 237 | 52 | 16 | 6 | 2 | 2 | - | 3 |
| MSG_A-2 | 7,510 | 797 | 175 | 42 | 10 | 4 | 2 | 2 | 1 | 5 |
| MSG_A-3 | 8,169 | 1,141 | 320 | 69 | 13 | 5 | 2 | 3 | 1 | 3 |
| MSG_M-1 | 7,703 | 1,055 | 291 | 61 | 23 | 5 | 5 | 1 | 1 | 2 |
| MSG_M-2 | 7,876 | 1,157 | 292 | 62 | 10 | 8 | 4 | - | 2 | 4 |
| MSG_M-3 | 8,118 | 1,173 | 311 | 68 | 16 | 11 | 1 | 1 | - | 4 |
| MSG_P-1 | 7,756 | 1,140 | 300 | 66 | 17 | 9 | 2 | 4 | 1 | 2 |
| MSG_P-2 | 7,997 | 1,083 | 281 | 74 | 21 | 4 | 3 | 1 | 1 | 3 |
| MSG_P-3 | 7,962 | 1,166 | 297 | 64 | 15 | 8 | 3 | 2 | - | 4 |
| MT-1 | 9,093 | 1,177 | 331 | 94 | 28 | 6 | 2 | - | 3 | 2 |
| MT-2 | 8,968 | 1,170 | 315 | 65 | 28 | 5 | 4 | 1 | - | 2 |
| MT-3 | 9,926 | 1,248 | 392 | 108 | 43 | 10 | 7 | 2 | 3 | 2 |
| OV-1 | 12,622 | 1,878 | 658 | 191 | 72 | 28 | 9 | 4 | 4 | 3 |
| OV-2 | 11,913 | 1,856 | 716 | 189 | 76 | 28 | 11 | 5 | 2 | 3 |
| OV-3 | 12,021 | 1,679 | 529 | 166 | 39 | 19 | 9 | 2 | - | 3 |
| PSG-1 | 7,344 | 1,053 | 250 | 52 | 10 | 8 | 1 | - | 1 | 2 |
| PSG-2 | 7,263 | 968 | 241 | 52 | 14 | 4 | 3 | - | - | 3 |
| PSG-3 | 7,580 | 994 | 253 | 47 | 14 | 3 | 1 | 1 | - | 2 |
| TT-1 | 11,650 | 1,921 | 688 | 208 | 79 | 24 | 10 | 9 | 1 | 4 |
| TT-2 | 12,383 | 2,017 | 746 | 238 | 104 | 30 | 12 | 9 | 3 | 5 |
| TT-3 | 11,671 | 2,122 | 763 | 255 | 96 | 36 | 18 | 8 | 4 | 4 |
| TT-3 | 11,671 | 2,122 | 763 | 255 | 96 | 36 | 18 | 8 | 4 | 4 |

Table S8. Number of tissue-specific expressed smORFs.

| Tissue | # smORF | |
| --- | --- | --- |
|  | TSI threshold=0.8 | TSI threshold=1.0 |
| ASG | 284 | 210 |
| FB | 294 | 221 |
| MG | 605 | 500 |
| MSG_A | 75 | 53 |
| MSG_M | 49 | 38 |
| MSG_P | 58 | 47 |
| MT | 532 | 401 |
| OV | 681 | 460 |
| PSG | 89 | 73 |
| SG | 517 | 365 |
| TT | 1702 | 1547 |
